# Event and Comment

**Published:** 1915-01

**Authors:** 


### DENTAL REGISTER

Vol. LXIX JANUARY 1915 No. 1

### EVENT AND COMMENT.

OHIO STATE DENTAL SOCIETY. The 49th Annual meeting of this Society was held in Columbus in Memorial Hall, December 1 to 3, 1914. The program was a good one, and the attendance was large and satisfactory. The new organization of all the local societies into the State Society has a material influence on the attendance and character of the meetings. In some respects the place of meeting was a great disappointment. The acoustics of the meeting hall were almost fatal to the literary program. The hall is too large and wholly unsuited to such a meeting, and we trust that some other arrangement will be made before another meeting is held there. It is not an ideal place either for holding clinics, as the light can not be controlled, and the arrangements were poor indeed. The exhibit space for dealers was very good, and the large number of dealers could find no fault with their accommodations. This part of the program was particularly attractive.

The program has already been published, and it was carried out as announced. None of the papers were on new subjects, and most of them had been presented subjectively before other societies. The most interest seemed to be in the subject of conductive anesthesia, as presented by Dr. Blum of New York. This was practically a restatement of the work of Guido Fisher, with the author's personal ideas additional. One evening session was devoted to a symposium on ''Allied Forces in Health Conservation." Some excellent addresses were made by the Governor of Ohio, President of the Ohio State University, President Ohio State Medical Association, Dr. H. C. Brown, a member of the Ohio Health Board, and others. This was a very profitable evening and demonstrated the great interest existing at present in all forms of public social service endeavors. It was interesting to notice that all the speakers had rather accurate knowledge of what the dental profession was doing in this direction, and what the prospects for real helpful co-operation appeared to be developing.

The clinics, as usual, were the most interesting and attractive features of the meeting. The special clinics were of the best kind and attracted great interest. The general clinics were difficult, as usual, to see, and it is hard to pass judgment on their worth, because no one could see all of them, as they were a large number (53) scheduled. Judging from the comments heard they were more than usually satisfactory. The ideal method and the ideal place for this kind of society work has not yet been devised, and it probably will not be until we have fewer clinics and more time in which to assimilate them. Socially the meeting seemed to be a success, although no effort was made by the local dentists to entertain the convention as a whole. Perhaps this is not best, but a general get-together around a banquet board always seems to tie folks together better than anything else, and it would serve to create a spirit of *esprit de corps* that would do much for the development of a united loyalty to the new form of organization that would be of great value. Would it not be worth while for the management to consider this idea when making up next year's program?

Dr. Price, Chairman of the Research Commission, made a very satisfactory report of the work done and congratulated the dentists of Ohio for the large share they had taken in this work.

Dr. E. C. Mills, of Columbus, was elected President for the ensuing year. Dr. Mills has done a great work the past year in perfecting the District organizations all over the State. Every section is now fully organized. Judging by Dr. Mills' past efforts and his tremendous energy we predict that the 1915 meeting of the Ohio State Society will be a record breaker.

WHAT OF 1915! With all scientific work abroad stopped because of the great war what is the prospect, professionally, for the new year? There will necessarily be suspension in all lines of activity not only abroad but at home. In America we shall have to apply ourselves more diligently to the scientific research problems, as we can not depend upon our German confreres for some time, if ever, to take up this work where they left it. The medical profession already realizes this and is taking steps to meet the emergency. It is a good time to encourage our Scientific Research Commission. It is not only our plain duty but our great opportunity.

THE FUTURE OF DENTAL EDUCATION. We are printing in this issue a paper read by Dr. G. V. I. Brown at the last annual meeting of the American Medical Association, on the influence of the rather close relationship of dentistry to medicine in modifying the contents of our dental curriculum in the near future. Because of the very nature of the service rendered by the dentist in the past, he has been compelled to know much of the fundamental sciences on which the practice of medicine is based. In these later times, owing to the increase in our knowledge of septic infection, we find there is still greater demand for scientific medical knowledge, that the technical practice of dentistry may be more conservatively and less perniciously engaged in by the profession. A year or so ago there seemed to be a general stampede for providing partially educated "technicers" to put into practice the hurriedly conceived ideals of preventive dentistry. The discussion thus aroused has culminated in a more thoughtful conception that something more than mere technical skill is demanded for permanent benefits along this line. College men especially, who are studying the subject, are naturally confronted with the problem in its practical details, and because of experience they are loth to undertake its adjustment in an educational way until they can solve it with satisfactory results from the scientific and pedagogic viewpoints. They already appreciate that it opens up new and still more intimate relations with medical knowledge and practice and that it means the adaptation of the principles of medical science to dental education and practice. There is scarcely a dental teacher who is not demanding more time for the teaching of his particular subject, and the time of the curriculum is already badly handicapped for lack of time in which to adequately present all the subjects of the course. And so we find there is a continuously growing sentiment that we must add another year to the course.

What shall be the content of this additional year seems to be one of the most troublesome factors. The reason for this is that each member of the faculty feels that more time should be given to his particular course, because of the continually broadening field for all kinds of professional services in the several departments. The management does not see how this additional emphasis can be put on the present courses, and other additional instruction be provided for the newer demands. How can we teach more dentistry and better dentistry, and at the same time introduce the newer and much to be desired medical subjects that seem destined to play important parts in future dental practice? At first we all say lengthen the course; then some one suggests that we employ better teachers and economize the time by more scientific pedogagy; and recently it is suggested in addition to both of these plans that we require higher standard of admission, such as a year or two of predental preparation in a good literary school. Now these are all excellent suggestions and there should be no objection to putting them into practice at once and the wonder is that it is not done. There are several reasons that we do not have to wait long to get, and the worst of it is they are so potent that they are practically sufficient to prevent the forward movement that every reasonably earnest student of this problem knows will come some time. They all involve the question of expense. It will cost more to make the kind of dentists we know we need and shall some day have, and where this factor enters most problems it dominates. If we require a student to spend one or two years in a literary school preparing himself to better assimilate the instruction in the sciences and arts of the dental curriculum, he objects because he can not command the necessary funds, and he does not care to lose the one or two years out of his life at that particular time. He is anxious to get at his job of serving the world in the sphere which he feels is calling for him. He can't be made to see that he will serve better and more truly if he is better prepared, and that there is real economy in making this preparation at the proper place and time in his career. Then the authorities who control the finances of the school can't see how it is possible for them to pay larger salaries to get superior instructors or more of them; or how they can afford better facilities even though it should result in facilitating and raising the quality of instruction. Most dental colleges are not money makers, and in all of them the teachers are underpaid, and are kept on their jobs because they have learned to love the work, and are ambitious to spend their lives in developing their calling to the higher ideals—which this kind of work engenders.

Another objection, which has some weight, is that owing to the fact that the colleges are not graduating sufficient students to do the work the people are now demanding, it would be very unwise to curtail the number of students entering college by making the course so rigid and expensive that few could afford to undertake it. Whatever may be said against this objection, it is all too real, and it has a powerful influence in restraining all movements to increase the cost of a dental education. We haven't space here to argue these propositions, but they are real factors in the problem that it will take time and strong influences of some kind to controvert. The profession may insist on legislation to bring it about, but it is more likely that continued agitation and the creation of a sentiment favorable to some advance in the dental as well as the medical profession will be the final controlling power. It is due and bound to come.

